# Contribution of large-pore channels to inflammation induced by microorganisms

**DOI:** 10.3389/fcell.2022.1094362

**Published:** 2023-01-09

**Authors:** José L. Vega, Camila Gutiérrez, Mauro Rojas, Juan Güiza, Juan C. Sáez

**Affiliations:** ^1^ Laboratory of Gap Junctions Proteins and Parasitic Diseases (GaPaL), Instituto Antofagasta, Universidad de Antofagasta, Antofagasta, Chile; ^2^ Centro de Investigación en Inmunología y Biotecnología Biomédica de Antofagasta (CIIBBA), Universidad de Antofagasta, Antofagasta, Chile; ^3^ Centro de Fisiología y Medicina de Altura (FIMEDALT), Universidad de Antofagasta, Antofagasta, Chile; ^4^ Centro Interdisciplinario de Neurociencias de Valparaíso (CINV), Instituto de Neurociencias, Universidad de Valparaíso, Valparaíso, Chile

**Keywords:** connexin, pannexin, innexin, LRRC8, CALHM, infectious disease

## Abstract

Plasma membrane ionic channels selectively permeate potassium, sodium, calcium, and chloride ions. However, large-pore channels are permeable to ions and small molecules such as ATP and glutamate, among others. Large-pore channels are structures formed by several protein families with little or no evolutionary linkages including connexins (Cxs), pannexins (Panxs), innexin (Inxs), unnexins (Unxs), calcium homeostasis modulator (CALHMs), and Leucine-rich repeat-containing 8 (LRRC8) proteins. Large-pore channels are key players in inflammatory cell response, guiding the activation of inflammasomes, the release of pro-inflammatory cytokines such as interleukin-1 beta (IL-1ß), and the release of adenosine-5′-triphosphate (ATP), which is considered a danger signal. This review summarizes our current understanding of large-pore channels and their contribution to inflammation induced by microorganisms, virulence factors or their toxins.

## 1 General Introduction

Plasma membrane ionic channels are necessary for fundamental cellular processes such as setting up resting membrane potentials, neuronal transmission, and the propagation of action potentials in electrically excitable cells (Bertil, 2001). Plasma membrane ionic channels selectively permeate ions (i.e., K^+^, Na^+^, Cl^−^, or Ca^2+^); However, large-pore channels are also permeable to small molecules, such as ATP, ADP, and NAD^+^, and glutamate, which contribute to physiological and pathophysiological responses (Kang et al., 2008; Cisterna et al., 2020; Syrjanen et al., 2021). Large-pore channels are structures formed by several protein families with no evolutionary linkages, including Cxs, Panxs, Inxs, Unxs, CALHMs, and LRRC8 proteins (Guiza et al., 2022). Despite little sequence homology, the large-pore channel members have similar transmembrane topologies with four transmembrane helices (Syrjanen et al., 2021).

Channels constituted by Cxs or Panxs are also termed hemichannels because they correspond to half of a gap junction channel (Orellana et al., 2012). The Cx gene family is present in vertebrates, and consists of 21 members in humans that differ by up to 29% in sequence identity (Laird & Lampe, 2018). Cx proteins form hexameric large-pore channels activated by proinflammatory cytokines, metabolic inhibition, depolarization, nitrosylation, dephosphorylation, and a divalent cation-free solution (Van Campenhout et al., 2021). The pannexin gene family is present in vertebrates, and consists of three members that differ by up to 75%–80% with Cxs in sequence identity (Panchin et al., 2000; Ruan et al., 2020). The Panx1 protein forms heptameric large-pore channels activated by phosphorylation by CAMKII, extracellular alkaline pH, and caspase cleavage (Penuela et al., 2013; Harcha et al., 2019; Lopez et al., 2021). Moreover, the Inx gene family is found exclusively in invertebrates and presents eight genes identified in *Drosophila melanogaster*, 25 in *Caenorhabditis elegans*, three in *Hirudo verdana*, and one gene in *Hydra polyps* (Guiza et al., 2018). Inx proteins form octameric large-pore channels activated by depolarization and mechanical stress (Guiza et al., 2018). Moreover, CALHM1 is a voltage- and Ca^2+^-gated channel that plays an essential role in the purinergic neurotransmission of sweet, bitter, and umami tastes (Taruno et al., 2013; Ma et al., 2016). CALHM1 form octameric large-pore channels in vertebrates, while the *Caenorhabditis elegans* CALHM1 assembles as non-amers, decamers, or undecamers (Demura et al., 2020; Ren et al., 2022). The VRACs are composed of LRRC8 proteins and are responsible for regulatory volume decreases after hypotonic cell swelling (Concepcion et al., 2022). Recent evidence suggests that kinetoplastid parasites have large-pore channel members formed by homologs of innexins, named unnexins, which seem to have a membrane topology similar to that of large-pore channels, and might play a critical role in infections (Guiza et al., 2022).

Cx hemichannels conduct K^+^, Na^+^, and Ca^2+^ (Mandal et al., 2015), and small molecules such as glutamate, glucose, NAD^+^, and ATP (Retamal et al., 2007; Anselmi et al., 2008; Okuda et al., 2013; Hansen et al., 2014). Panx1 hemichannels also permeate Cl^−^, ATP and glucose (Ma et al., 2012; Riquelme et al., 2013). VRAC channels conduct Cl^−^ and other halide ions, but also transport a variety of organic molecules such as taurine, inositol, glutamate, as well as therapeutic agents such as cisplatin, carboplatin, and blasticidin S, and immunomodulatory cyclic dinucleotides such as 2′3′cGAMPs (Concepcion et al., 2022). Of particular interest for this review are channels formed by Cx43, CALHM1, Panx1 and innexins, which are permeable to ATP and Ca^2+^ ions (Locovei et al., 2006; Kang et al., 2008; Schalper et al., 2010; Ma et al., 2016; Shan et al., 2020). Because ATP externalization is the first step in the cascade of events leading to the maturation and secretion of IL-1ß and IL-18, multiple studies have proposed that large-pore channels play a relevant role in inflammation (Makarenkova and Shestopalov, 2014; Sáez and Green, 2018). Accordingly, blocking large-pore channels attenuates inflammation and prevents cell death (Makarenkova and Shestopalov, 2014; Sáez and Green, 2018).

## 2 Modulation of large-pore channels by bacteria and pathogen-associated molecular patterns and their role in bacterial diseases

Peptidoglycans (PGNs) are a cell wall component of Gram-positive bacteria considered to be a pathogen-associated molecular patterns (PAMPs) that can promote the generation of pro-inflammatory cytokines, such as tumor necrosis factor-alpha (TNF-alpha) and IL-1ß, which promote a systemic inflammatory response by activating Toll-like receptor two signaling (Amoureux et al., 2005; Robertson et al., 2010). Interestingly, PGNs can modulate large-pore channels (Robertson et al., 2010). For example, a PNG derived from *Staphylococcus epidermidis* (strain NCIMB 40896) has been shown to increase Cx43 hemichannel activity in HeLa-Cx43 cells, which can be prevented by LnCl_3_, carbenoxolone or Gap26 (Robertson et al., 2010). Moreover, carbenoxolone prevents the induction of IL-6 and TLR2 mRNA expression induced by PGN (Robertson et al., 2010).

Lipopolysaccharides (LPSs) of Gram-negative bacteria are potent proinflammatory PAMPs that can induce the expression of Cx43 or activate large-pore channels, hence promoting inflammation (Eugenin et al., 2001; Eugenin et al., 2003; Chae and Bothwell, 2019; Huang et al., 2020; Ma et al., 2020). For example, carbenoxolone (dose of 20 mg/kg for 30 min before LPS injection) has been shown to reduce the production of IL-1β, IL-6, and TNF-α, as well as tubular cell apoptosis in a model of sepsis-induced acute kidney injury (Huang et al., 2020). Silencing Panx1 was observed to decrease inflammatory cytokine production, apoptosis, NLRP3 inflammasome activation, and pro-apoptosis in LPS-treated HK-2 cells (Huang et al., 2020). Selective inhibition of Cx43 hemichannels also protects HUVEC cells from LPS-induced apoptosis (Ma et al., 2020). LPS from *Escherichia coli* (*E. coli)* (serotype O111:B4) increases Cx43 levels in the plasma membrane of HUVECs, and treatment with Gap19 reduces LPS-induced intracellular ROS and apoptotic levels in HUVECs (Ma et al., 2020). Along the same line of analysis, LPS (from *E. coli* serotype O111:B4) was seen to increase ethidium uptake in microglia, which can be blocked by probenecid, ^10^Panx1, and siRNA for Panx1 (Orellana et al., 2013a). Yet another study showed that LPS increases astroglial Cx43 hemichannel activity in acute hippocampal slices (Abudara et al., 2015). The LPS-induced Cx43 hemichannel activation in astrocytes is mediated, to a great extent, by pro-inflammatory cytokines released from activated microglia (Retamal et al., 2007). Prenatal exposure to LPS increases Cx43 and Panx1 hemichannel activity in reactive astrocytes in offspring (Avendano et al., 2015). In the periphery, LPS causes severe muscle deterioration due to higher sarcolemma permeability, and a decline in resting membrane potential (Cea et al., 2019).

In 2019, we demonstrated that mice treated for 5 h with LPS (from *E. coli*) induced the appearance of functional Cx hemichannels in myofibers freshly isolated from skeletal muscle (Cea et al., 2019). These results suggest that sarcolemmal dysfunction induced by endotoxemia is partially due to *de novo* expressions of functional Cx43-and Cx45-formed large-pore channels, which are also expressed in skeletal muscles during sepsis (Balboa et al., 2018; Cea et al., 2019). Interestingly, LPS-induced neuroinflammation was remarkably less in microglia from CALHM2 knock-out mice, suggesting the participation of microglia CALHM2 channels in the neuroinflammation produced by LPS (Cheng et al., 2021).

*Shigella flexneri* (*S. flexneri*) is the causative agent of bacillar dysentery, causing intestinal inflammation (Tran Van Nhieu et al., 2003). *S. flexneri* induces the activation of caspase-1, leading to pyroptotic cell death in macrophages (Suzuki et al., 2014). It is noteworthy that *S. flexneri* modulates large-pore channels to favor its spread and invasion (Tran Van Nhieu et al., 2003; Bonnet and Tran Van Nhieu, 2016). For example, *S. flexneri* (M90T strain) increases Lucifer yellow uptake in HeLa-Cx26 cells, whereas challenging with the non-invasive mxiD mutant strain induced minimal dye incorporation (Tran Van Nhieu et al., 2003). In addition, *S. flexneri* induces ATP release in HeLa-Cx26 cells—a response blocked by carbenoxolone, which is a derivative of 18-α-glycyrrhetinic acid (Tran Van Nhieu et al., 2003). Treatment with 18-α-glycyrrhetinic acid consistently reduced the number of infected cells per dissemination focus in HeLa-Cx26 (Tran Van Nhieu et al., 2003). Furthermore, Cx26 hemichannels facilitate gastrointestinal bacterial infection caused by *E. coli* (Simpson et al., 2013). A significant reduction in both cellular invasion and adherence by *E. coli* (E69 strain) was also demonstrated in human intestinal cell lines (Caco-2 and HT-29 cells) following treatment with Cx26 siRNA (Simpson et al., 2013). Moreover, the R143W Cx26 mutant causes a reduction in *E. coli* adherence (Simpson et al., 2013). Another study showed that Cx43 hemichannels are involved in the pathogenesis of *Yersinia enterocolitica* (*Y. enterocolitica*), which is a Gram-negative pathogen that causes a broad range of gastrointestinal syndromes (Velasquez-Almonacid et al., 2009). HeLa-Cx43 cells challenged with *Y. enterocolitica* resulted in higher bacterial uptake than parental cells (Velasquez-Almonacid et al., 2009). *Y. enterocolitica* also increased Lucifer yellow uptake, a response blocked by carbenoxolone in HeLa-Cx43 cells (Velasquez-Almonacid et al., 2009). Similarly, endotoxemia induces *de novo* expression of Cxs and upregulates Panx1 in skeletal muscles, where they are likely to form hemichannels (Balboa et al., 2018). *Clostridioides difficile* (*C. difficile*) is a Gram-positive, anaerobic toxin-producing *bacillus* that causes nosocomial diarrhea associated with antibiotic use (Loureiro et al., 2022). Infecting C57BL/6 mice with *C. difficile* (VPI10463 strain) was shown to increase levels of Panx1 in the cecum and colon (Loureiro et al., 2022). Blocking Panx1 with mimetic peptide ^10^Panx1 was observed to decrease caspase-3/7 activity and phosphatidylserine-annexin-V binding in toxin A- and toxin B-challenged enteric neurons and enteric glial cells (Loureiro et al., 2022).

*Streptococcus pneumoniae,* which is a major causative agent of bacterial meningitis, was shown to increase astroglial Cx43 hemichannel activity (Bello et al., 2020) (Table 1). The authors explained that purified pore-forming toxin pneumolysin promotes the Cx43-dependent release of extracellular ATP, and prolongs the increase of cytosolic Ca^2+^ in host cells (Bello et al., 2020).

**TABLE 1:** Key publications describing the functional regulation of large-pore channels by pathogens.

Pathogens	Large-pore channels	Mechanisms	Cell types	Reference
Parasites				
*Trypanosoma cruzi*	Opening Panx1	ATP release, P2Y_1_ activation, increase of cytosolic Ca^2+^	Cardiomyocytes	Barria et al. (2018)
Virus				
*HIV*	Opening Panx1	ATP release	T Lymphocytes	Orellana et al. (2013b).
*HIV*	Opening Cx43	DKK1 release	Astrocytes	Orellana et al. (2014)
*SARS-Cov-2*	Opening Panx1	ATP, PGE_2_ and IL-1ß release, *via* P2X_7_ activation	Lung epithelial cells	Luu et al. (2021)
Bacterias				
*Shigella flexneri*	Opening Cx26	ATP release	Epithelial cells (Caco-2/TC7)	Tran van nhieu et al. (2003)
*Streptococcus pneumoniae*	Opening Cx43	ATP release, and increase of cytosolic Ca^2+^	Astrocytes	Bello et al. (2020)
*Yersinia enterocolitica*	Opening Cx43	Tyrosine phosphorylation of Cx43	Hela-Cx43	Velasquez-Almonacid et al. (2009)
*Clostridioides difficile*	Opening Panx1	ATP release, P2X_7_ activation, release of caspase 3/7, and IL-6	Enteric glial cells	Loureiro et al. (2022)

HIV, human immunodeficiency virus; SARS-Cov-2, severe acute respiratory syndrome coronavirus 2.

Finally, bacteria have been shown to use innexin-formed large pore channels for their mechanism of infection in insects. For example, *Vibrio alginolyticus*, *Vibrio parahaemolyticus*, or LPS (from *E. coli* serotype O55:B5) can induce upregulation of Inx2 gene expression in hemocyte, gill, and hepatopancreas tissues in *Scylla paramamosain* (Wang et al., 2015). The authors indicated that ectopic expression of Sp-inx2 in HeLa and epithelioma papulosum cyprinid cells can induce apoptosis (Wang et al., 2015).

## 3 Modulation of large-pore channels by virus, and their role in viral diseases

Human immunodeficiency virus (HIV) causes a public health problem, and has claimed more than 35 million lives worldwide (Malik and Eugenin, 2019). HIV can modulate Cx43 or Panx1 hemichannels (Orellana et al., 2013b; Orellana et al., 2014). For example, HIV induces the opening of Panx1 hemichannels in CD4^+^ T lymphocytes (Orellana et al., 2013b). The ^10^Panx1 mimetic peptide inhibits HIV replication in CD4^+^ T lymphocytes (Orellana et al., 2013b). In a subsequent study, it was demonstrated that HIV causes the opening of Cx43 hemichannels, an effect that can be blocked by lanthanum ions, Cx43E2 antibodies, or mimetic peptide gap26 (Orellana et al., 2014). Another study showed that the simian immunodeficiency virus (SIV) causes Panx1 hemichannels to open in peripheral blood mononuclear cells isolated from SIV-infected macaques (Gorska et al., 2021). A subsequent study demonstrated that peripheral blood mononuclear cells isolated from HIV-infected individuals have a spontaneous opening of Panx1 hemichannels, which results in increased circulating ATP levels and prostaglandin E2 in the serum of all HIV-infected individuals (Velasquez et al., 2020). Moreover, the protein S from severe acute respiratory syndrome coronavirus 2 (SARS-CoV-2) induces a transient increase in ethidium uptake in human lung epithelial cells. This effect was blocked by probenecid or ^10^Panx1 peptide, suggesting the involvement of open Panx1 hemichannels. It should be noted that blocking Panx1 hemichannels reduces viral entry and replication in human lung epithelial cells, suggesting a critical role for Panx1 in SARS-CoV-2 infections (Luu et al., 2021) (Table 1). Moreover, the herpes simplex virus infection causes necrotic cell death in murine embryonic fibroblasts, a process that is often inversely correlated with an interferon response (Zhou et al., 2020). LRRC8A^−/−^ cells were observed to exhibit higher viral loads after HSV-1 (KOS strain) infection, suggesting that VRACs participate in the propagation of the herpes simplex virus (Zhou et al., 2020). A comprehensive review of the structure and possible mechanisms underlying pore inhibition and modulation by targeting the intracellular leucine-rich repeat (LRR) domain has been recently reported (Pereira da silva et al., 2022).

## 4 Modulation of large-pore channels by parasites, and their role in parasitic diseases

*Trypanosoma cruzi* (*T. cruzi*) is a kinetoplastid parasite that causes Chagas disease in humans, which is characterized by severe cardiomyopathy and gastrointestinal motility disorders (Adesse et al., 2011). *T. cruzi* can also modulate intercellular communication *via* gap junctions in cardiac myocytes, brown adipocytes, astrocytes, and leptomeningeal cells (de Carvalho et al., 1992; Campos de Carvalho et al., 1998; Burke et al., 2014). Recently, we described that *T. cruzi* could modulate large-pore channels. Exposure to *T. cruzi* (H510 strain) was observed to increase Panx1 hemichannel activity in Hela-Panx1 (Barría et al., 2018). *T. cruzi* or supernatants from *T. cruzi* cultures (epimastigotes) also increase ethidium uptake in neonatal rat cardiac myocytes (Barría et al., 2018). ^10^Panx1 or probenecid prevents a *T. cruzi*-induced [Ca^2+^]_*i*_ transient as well as *T. cruzi* invasion (Barría et al., 2018) (Table 1).

*Leishmania* is a protozoan parasite that causes leishmaniasis (Alvar et al., 2012). The three main clinical manifestations are cutaneous, mucocutaneous, and visceral leishmaniasis (Alvar et al., 2012). The *Leishmania amazonensis* (*L. amazonensis*) infection upregulates expression and function of the P2Y_2_ receptor in macrophages (Marques-da-Silva et al., 2011). Treatment with uridine triphosphate (UTP, P2Y_2_ agonist) reduces parasite load and triggers apoptosis in macrophages infected with *L. amazonensis* (Marques-da-Silva et al., 2011). The effect of apoptosis induced by UTP was not observed in *L. amazonensis*-infected macrophages from Panx1^−/−^ mice (Thorstenberg et al., 2018). The authors suggest that the Panx1 and the P2X_7_ receptors would be involved in controlling *L. amazonensis* infection induced by uridine triphosphate treatment (Marques-da-Silva et al., 2011; Thorstenberg et al., 2018).

## 5 Conclusion and futures

The inflammatory process is a complex and multistep process, requiring the recruitment of various cells, such as fibroblasts, glial cells (Seo et al., 2021), endothelial cells (Kameritsch and Pogoda, 2020), resident leukocytes (Kameritsch and Pogoda, 2020), and mast cells, among others (Medzhitov, 2008; Medzhitov, 2021). In addition, most cells involved in inflammation express functional large-pore channels in their plasma membrane, where they play critical roles in inflammatory responses, mainly as exit routes for ATP in inflamed cells (Crespo Yanguas et al., 2017; Syrjanen et al., 2021). Upon ATP release, several enzymes degrade ATP into ADP, AMP, and adenosine, thus amplifying the response, given that they also signal through purinergic receptors (Fredholm et al., 1994; Ralevic and Burnstock, 1998; Velasquez and Eugenin, 2014). The importance of purinergic signaling in inflammation has been established in the last decades (see the following reviews: Idzko et al., 2014; Cekic and Linden, 2016; Dosch et al., 2018). However, the role of large-pore channels and purinergic signaling in inflammation generated by infectious disease has only recently been examined (Eugenin, 2014; Velasquez and Eugenin, 2014).

**FIGURE 1:**
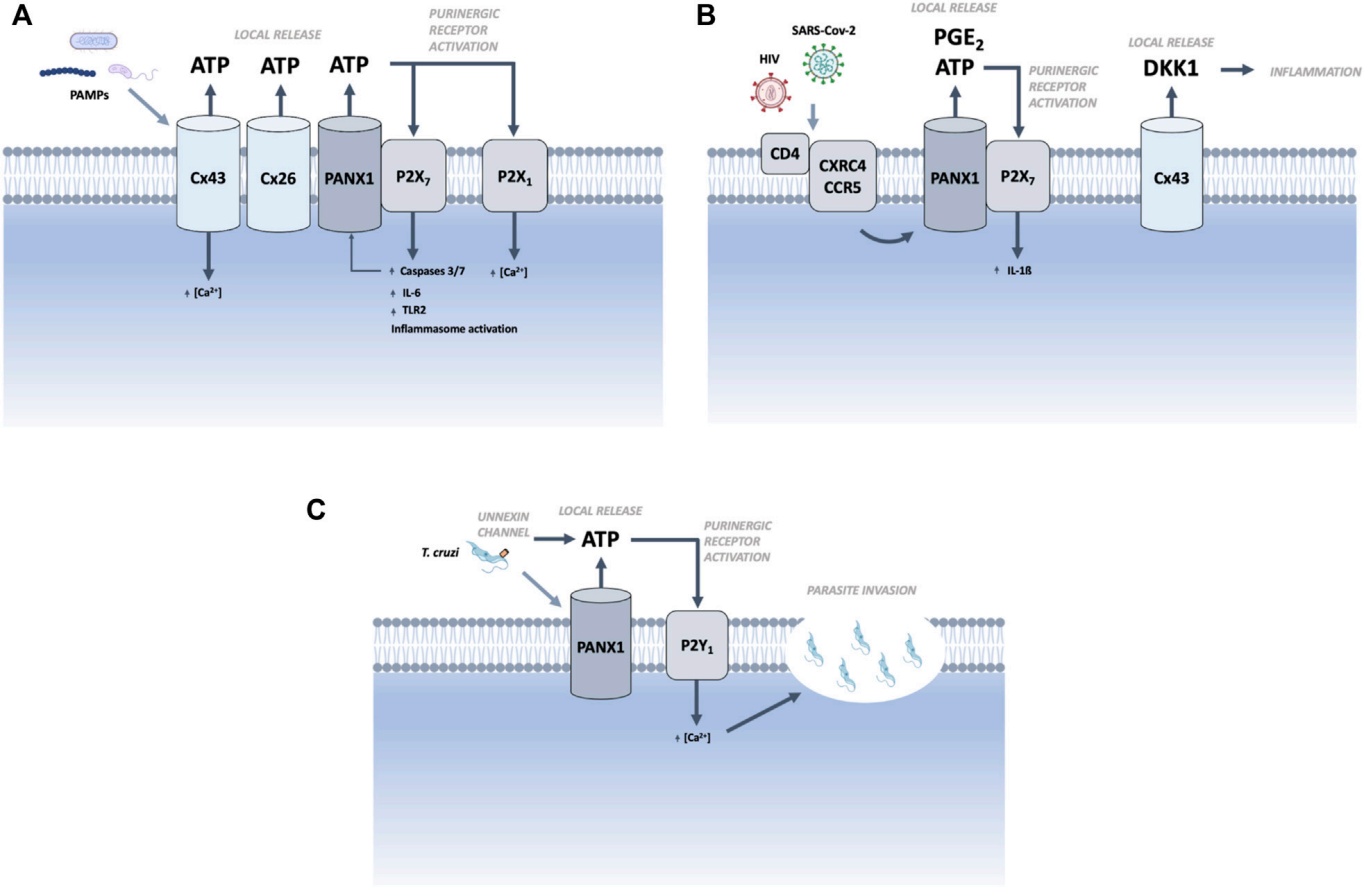
Proposed mechanism of contributions of large-pore channels to inflammation induced by microorganisms. **(A)** Extracellular bacteria or their toxins induce opening of Cx43, Cx26, and Panx1 hemichannels in host cell, causing an outflow of ATP. Extracellular ATP binds to specific purinergic receptors, enhancing inflammation. The opening of the Cx43 hemichannel allows for the entry of Ca^2+^. **(B)** Through the activation of CD4-CXCR4/CCR5, the virus induces the opening of Cx43 and Panx1 hemichannels in the host cell, causing an outflow of ATP, PGE_2_, and DKK1. **(C)** Trypanosomatids induce the opening of Panx1 hemichannels in the host cells, causing local ATP release. Through the activation of the P2Y_1_ receptor, ATP increases intracellular calcium ions and consequent invasion. The presence of hemichannels formed by unnexins in the parasite could be a tentative source of ATP. Figure was created with biorender.com.

In bacterial infections, PAMPs activate hemichannels formed by Cx43, Cx26, or Panx1, causing an outflow of ATP. The latter, through the activation of P2X_7_ receptors, induces the activation of caspase 3/7 (Loureiro et al., 2022) and the production of IL-6 (Robertson et al., 2010; Loureiro et al., 2022). By activating P2X_1_ receptors, it can increase the concentration of cytosolic Ca^2+^ ions (Wang et al., 2017). Moreover, Panx1 is a target for caspases 3 and 7, whose activation results in a constitutively open channel and, therefore, more ATP release, which generates a feedforward mechanism that could be blocked by using Panx1 hemichannels or P2X receptor blockers (Chekeni et al., 2010; Narahari et al., 2021) (Figure 1A).

In viral infections, SARS-CoV-2, hCoV-229E, or HIV activate Panx1 hemichannels *via* CD4/CXCR4 CD4/CCR5, causing an outflow of ATP, which induces the maturation of IL-1ß through the activation of P2X_7_ receptors (Orellana et al., 2013a; Luu et al., 2021) (Figure 1B). HIV can activate Cx43 hemichannels, inducing the release of dickkopf-1 (DKK1) proteins, which play a pivotal role in pathological inflammatory diseases (Orellana et al., 2014; Cae et al., 2019; Park et al., 2022). Therefore, Panx1 hemichannels blocker might be effective in treatments to reduce viral load.

In parasite infections, T. cruzi or *T. cruzi*-virulence factors have been described to activate Panx1 hemichannels in cardiac cells, causing an outflow of ATP, and increasing the concentration of cytosolic Ca^2+^ through the activation of P2Y_1_ receptors, which is necessary for parasite invasion (Barria et al., 2018) (Figure 1C). Recent evidence suggests the presence of members of large-pore channels in T. cruzi, which could be a route for ATP release, and could be considered for future research (Güiza et al., 2022).
